# Characterization, Modeling and Design Parameters Identification of Silicon Carbide Junction Field Effect Transistor for Temperature Sensor Applications

**DOI:** 10.3390/s100100388

**Published:** 2010-01-05

**Authors:** Tarek Ben Salah, Sofiane Khachroumi, Hervé Morel

**Affiliations:** 1 Ampere, CNRS UMR 5005, INSA de Lyon, bâtiment Léonard de Vinci, 69621 Villeurbanne, France; E-Mail: herve.morel@insa-lyon.fr; 2 Electrical System Laboratory, UR03ES05, ENIT, Tunis, BP 37, le Belvédère, 1002 Tunis, Tunisia; E-Mail: sofianekha204@gmail.com

**Keywords:** silicon carbide, SiC-JFET sensor, modelling, design parameters

## Abstract

Sensor technology is moving towards wide-band-gap semiconductors providing high temperature capable devices. Indeed, the higher thermal conductivity of silicon carbide, (three times more than silicon), permits better heat dissipation and allows better cooling and temperature management. Though many temperature sensors have already been published, little endeavours have been invested in the study of silicon carbide junction field effect devices (SiC-JFET) as a temperature sensor. SiC-JFETs devices are now mature enough and it is close to be commercialized. The use of its specific properties *versus* temperatures is the major focus of this paper. The SiC-JFETs output current-voltage characteristics are characterized at different temperatures. The saturation current and its on-resistance *versus* temperature are successfully extracted. It is demonstrated that these parameters are proportional to the absolute temperature. A physics-based model is also presented. Relationships between on-resistance and saturation current *versus* temperature are introduced. A comparative study between experimental data and simulation results is conducted. Important to note, the proposed model and the experimental results reflect a successful agreement as far as a temperature sensor is concerned.

## 1. Introduction

Future semiconductor devices should have the ability to work in harsh environments. Indeed, neither high temperature nor aggressive chemical application demands can be fulfilled by devices based on silicon semiconductor technology [1–3]. Silicon carbide (SiC) presents an alternative that can be applied as an active material for sensors in extreme environments like turbines engines, geothermal wells, among many others [4,5]. Much attention has been given to SiC semiconductor on account of its physical and electrical properties [6,7]. Contrasted with silicon, SiC has a higher breakdown electric field, a higher electron saturation velocity, a higher thermal conductivity and a larger band gap. The large band gap allows high temperature operation up to 1,200 K in chemically reactive environments [8]. Though many sensors devices have already been published, little efforts have been investigated to study Silicon Carbide Junction Field Effect sensor (SiC-JFETs) as a temperature sensor. This paper presents an experimental investigation of SiC-JFET. Static modes at different temperatures is presented. The variation of the saturation current, I_DSS_, and the output drain to source resistance, R_DSon_, *versus* temperature are successfully extracted. It is experimentally demonstrated that these parameters are proportional to the absolute temperature for large range with a minor linearity error. Parameter such as linearity is proved to be of vital importance for many industrial applications. Crucially though, SiC-JFETs modelling approaches are under-researched in literature [8–12].

One promising model is proposed in [11]. Such a model, though declared accurate, does not adequately present the output transfer characteristics of the SiC-JFET [12]. Further, the standard Spice model is found to be incapable of correctly predicting both the two linear and saturation regions in the static mode in a unified manner. Another limitation is that this model is unsatisfactory at high temperature [12]. So far, SiC-JFETs temperature dependant physically-based model issues has not been widely discussed in literature. Hence, improving JFET models with more precision is the core purpose of this paper. Particular attention is set out to identify the SiC-JFET design parameters. It is difficult to obtain these parameters directly from the manufacturer. Design parameters are crucial for sensor behaviour, modelling and fabrication procedure.

The present paper falls into four sections. It unfolds with a section about the modelling of the SiC-JFETs *versus* temperature. This model, run for a wide range of temperature, is based on the physical and the behavioural analysis of the JFET. Section 3 introduces the experimental investigations of the SiC-JFET. The variation of the saturation current and the on-resistance *versus* temperature are also discussed for many SiC-JFETs. Section 4 dwells upon validation. A comparative study between experimental and simulation is undertaken.

## 2. Model Development

**Figure 1.**
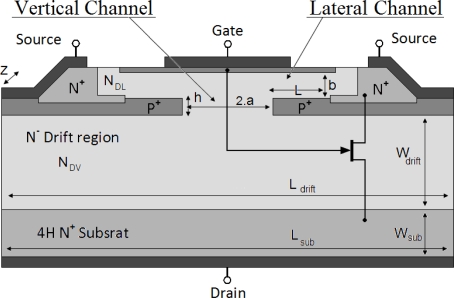
Cross-sectional view of the two channel SiC-JFET structure.

In Figure 1 the structure of a half cell of the studied two-channel SiC-JFET is shown. The lateral channel is sandwiched between p gate section at the middle of the device and p+ buried gate sections spread to both ends. The vertical channel is located between the two p+ regions. In Figure 1, N_DL_ is the doping concentration in the lateral channel, L is the lateral channel length, b is the lateral channel width, h is the vertical channel length, 2a is the vertical channel width, N_DV_ is the doping concentration in the drift region and in the vertical channel, W_Drift_ is the width of the drift region, L_Drift_ is the width of the active cell, W_Sub_ is the width of the substrate region and Z is the device width.

**Figure 2.**
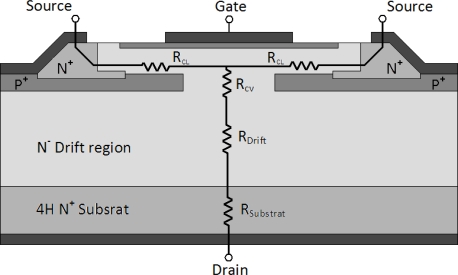
Two channels SiC-JFET resistance model.

The two-channel structure exploits the drift region which has the main contribution to the on-state resistance (Figure 2). In Figure 2a schematic resistive is presented. This resistance is a major concern for the device engineers because it determines the conduction losses and imposes a voltage drop at the boundaries of the component. The lower the on-resistance is, the less losses are. The specific on-resistance can be calculated by Equation (1):

(1) $$R_{{\mathrm{DS}}_{\mathrm{ON}}}=\frac{R_{\mathrm{CL}}}{2}+R_{\mathrm{CV}}+R_{\mathrm{Drift}}+R_{\mathrm{Sub}}$$

where *R_CL_*, given by Equation 2 is the lateral channel resistance. The calculation of this resistance comes from the geometrical parameters of the lateral channel.

(2) $$R_{\mathrm{CL}}=\frac{L}{q.N_{\mathrm{DL}}.\mu_{n}.Z.b}$$

The *R_CV_* is the vertical channel resistance without any bias given by Equation (3):

(3) $$R_{\mathrm{CV}}=\frac{h}{2.q.N_{\mathrm{DL}}.\mu_{v}.Z.a}$$

The drift region resistance is represented as the resistance of a rectangular area in the drift region under the channel. The drift resistance is given by Equation (4):

(4) $$R_{\mathrm{Drift}}=\frac{W_{\mathrm{drift}}}{q.N_{\mathrm{DV}}.\mu_{v}.Z.L_{\mathrm{drift}}}$$

The substrate resistance is calculated with the same Equation 4. The substrate resistance is given by Equation (5):

(5) $$R_{\mathrm{Sub}}=\frac{W_{\mathrm{Sub}}}{q.N_{\mathrm{Sub}}.\mu_{\mathrm{Sub}}.Z.L_{\mathrm{Sub}}}$$

where *q* is the electron charge and *μ* is the electron mobility in the channel given by [13,14]:

(6) $$\mu =\frac{947}{1+{\left(\frac{N_{\mathrm{DL}}}{{1.94e}^{23}}\right)}^{0.61}}.{\left(\frac{T}{300}\right)}^{-2.15}$$

**Figure 3.**
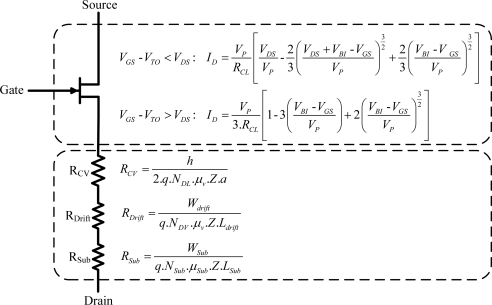
The two channels SiC-JFET circuit model.

The specific on-resistance is a strong function of the mobility. It should be noted that the JFET on-resistance depends also on the technological parameters: The doping concentration, (*N_DL_*, *N_DV_* and *N_sub_*) and the width (*W*, *h* or *L*) (Equations 1–5). An optimal couple of the doping concentration and the width of the JFET guarantee a minimum on-resistance and power loss. The JFET lateral-region current expression [13] Figure 3, found by integrating the resistive voltage drop along the channel while taking into account the variation of the channel-width b, can be written in the following form:

(7) $$I_{D}=\frac{V_{P}}{R_{\mathrm{CL}}}\left[\frac{V_{\mathrm{DS}}}{V_{P}}-\frac{2}{3}{\left(\frac{V_{\mathrm{DS}}+V_{\mathrm{BI}}-V_{\mathrm{GS}}}{V_{P}}\right)}^{\frac{3}{2}}+\frac{2}{3}{\left(\frac{V_{\mathrm{BI}}-V_{\mathrm{GS}}}{V_{P}}\right)}^{\frac{3}{2}}\right]\;\text{for}\;V_{\mathrm{GS}}-V_{\mathrm{TO}}<V_{\mathrm{DS}}$$

where *V_BI_* is the built-in potential and *V_p_* is the pinch-off voltage given by:

(8) $$V_{P}=\frac{q.N_{\mathrm{DL}}.b^{2}}{2.\varepsilon_{\mathrm{Sic}}}$$

where ε_SiC_ is the permittivity of the SiC semiconductor material.

The saturation-region current expression is obtained by finding the voltage *V_DS_* for which the derivative of Equation (7) with respect to *V_DS_* equals zero:

(9) $$I_{D}=\frac{V_{P}}{3.R_{\mathrm{CL}}}\left[1-3\left(\frac{V_{\mathrm{BI}}-V_{\mathrm{GS}}}{V_{P}}\right)+2{\left(\frac{V_{\mathrm{BI}}-V_{\mathrm{GS}}}{V_{P}}\right)}^{\frac{3}{2}}\right]\;\text{for}\;V_{\mathrm{GS}}-V_{\mathrm{TO}}>V_{\mathrm{DS}}$$

Note that this model is based on the physical and behavioural analysis of the JFET, taking into account the two channels and the influence of the temperature.

## 3. Simulation Results

The proposed model (Figure 3), described by Equations 1–9, is implemented in VHDL-AMS (defined by IEEE Std.1076.1 in 1999) [15] and simulated in the Simplorer simulator (by Ansoft/ANSYS). Additional details about VHDL-AMS are described in [16].

**Figure 4.**
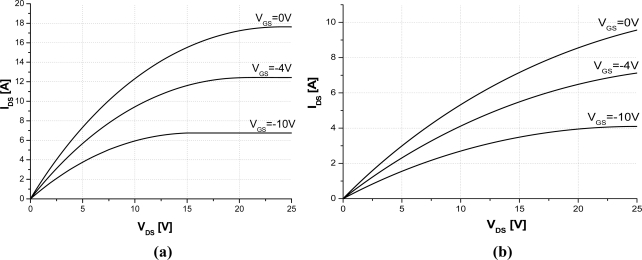
Simulation DC characteristics of the studied two channels SiC-JFET. (a) 300 K and (b) 500 K. (a = 0.46 μm, b = 0.76 μm, L = 5.7 μm, h = 1.05 μm, N_DL_ = 5 × 10^16^ cm^−3^, N_DV_ = 1.3 × 10^16^ cm^−3^, W_drift_ = 7.5 μm, L_drift_ = 19 μm, Z = 3.8 cm).

Figure 4 presents the DC simulation results of the studied two-channel SiC-JFET sensor at room and 500 K temperature. Figure 4 demonstrates that the saturation current decrease and the on-resistance increase *versus* temperature. It is, however, interest to depict the behavior of these parameters at different temperature.

**Figure 5.**
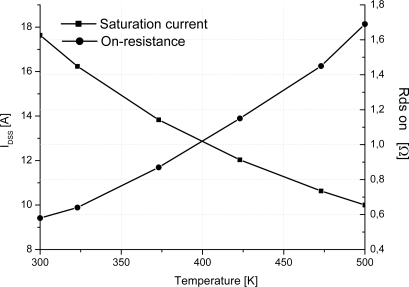
On-resistance and saturation current *versus* temperature of the studied two channels SiC-JFET (a = 0.46 μm, b = 0.76 μm, L = 5.7 μm, h = 1.05 μm, N_DL_ = 5 × 10^16^ cm^−3^, N_DV_ = 1.3 × 10^16^ cm^−3^, W_drift_ = 7.5 μm, L_drift_ = 19 μm, Z = 3.8 cm).

Figure 5 depicts the variation of the on-resistance *versus* temperature. On-resistance is proportional to the absolute temperature in the 300–500 K temperature range. Figure 5 gives also the variation of the saturation current *versus* temperature. Similar to the on-resistance model, the saturation current is proportional to the absolute temperature. A second important result is then proved for SiC-JFET giving this component more capabilities to be applied in many applications amid temperature sensor. Consequently, it is important to note that the ability of the JFET-based SiC semiconductor to operate at high ambient temperature is widely better than silicon device counterpart.

## 4. SiC-JFET Characterization

In this section, SiC-JFET is experimentally characterized for both room and high temperatures. On-state resistance and saturation current are extracted at different temperature.

### 4.1. Room Temperature Characterization

**Figure 6.**
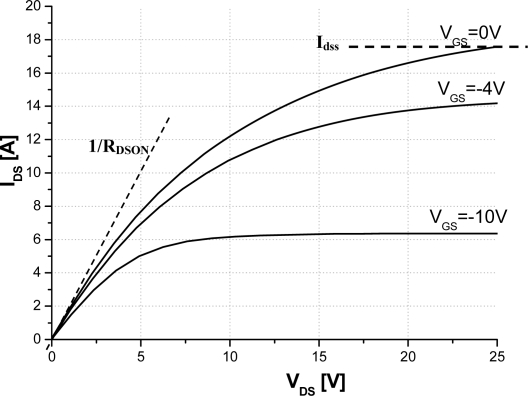
Experimental drain-current *versus* drain voltage characteristics as a Vgs parameter at room temperature of the 15 A-1,200 V SiC-JFET sample.

To measure the SiC-JFET transistor in a steady state, a positive bias between drain and source is used while the gate voltage is decreased from 0 down to the pinch-off voltage. The applied voltages on the drain, V_DS_, are selected to evidence the two different regions (linear and saturation). These regions are featured by on-resistance and saturation current when *V*_GS_ = 0 V. Figure 6 is an illustration of the steady state electrical characterizations in forward mode as a *V*_GS_ parameter. Curve tracer Tektronix 371A is used for these characterizations. Pulse width is to be fixed at 250 μs if the current magnitude is important so as to sidestep self-heating.

### 4.2. High Temperature Characterization

**Figure 7.**
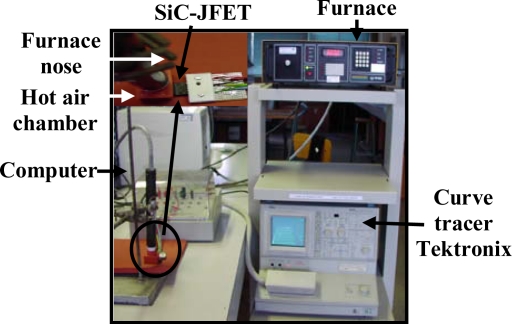
Experimental test bench for high temperature static characterisation of a SiC-JFET. The data acquisition is done via a RS232 to a computer for data display and storage.

**Figure 8.**
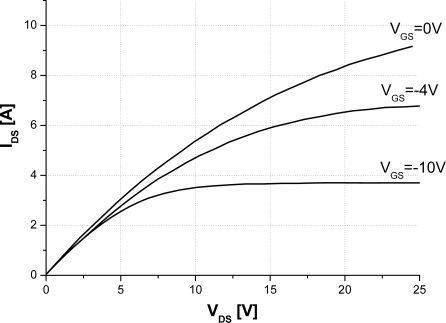
Experimental drain-current *versus* drain voltage characteristics as a Vgs parameter at 500 K of the 15 A-1,200 V SiC-JFET sample.

Theoretically speaking, a SiC semiconductor can work at high ambient temperature compared to the silicon semiconductor counterpart. Due to the limitations in the packaging, realizing operations at very high temperatures is difficult. A case in point, polymer packaging is excluded from high ambient temperature. Low temperature co-fired ceramic is, on the other hand, deemed one of the promising types of packaging for SiC devices. Indeed, it can considerably decrease the current handling capability. On the basis of this background, very high temperature applications will have to be delayed until the packaging evolves. Characterization until 500 K is, therefore, possible for TO3 and TO2 packages [17,18]. For temperature measurement, the experimental circuit in Figure 7 is used. This circuit is equipped with a thermal management unit TP014H that controls the temperatures of the JFET under test. The highest temperature measurement errors are 3 °C Using this system along with a curve tracer (Figure 7), the SiC-JFET out-put transfer characteristics at different temperature are obtained. Figure 8 depicts the SiC-JFET forward mode behaviour at 500 K. As a result, the saturation current decreases and the on-resistance increases at high temperature. Therefore, it is interesting to experimentally study the variation of the saturation and the on-resistance for many temperature levels.

In the following, the on-resistance is extracted from the slope of the out-put transfer characteristic by a zero gate-source bias when drain-source bias moves to zero as illustrated in the consequent formula:

(11) $$R_{\mathrm{ON}}={\frac{1}{\frac{\partial I_{D}}{\partial V_{\mathrm{DS}}})}}_{\left(V_{\mathrm{GS}}=0,V_{\mathrm{DS}}=0\right)}$$

**Figure 9.**
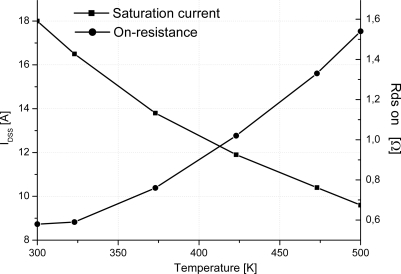
Experimental on-resistance and saturation current *versus* temperature of the 15 A-1,200 V SiC-JFET sample.

The saturation current is also obtained directly from the out-put transfer characteristic when V_GS_ = 0 V. The saturation current and the on-resistance are shown in Figure 9. Both of them give an abacus of these electrical parameters *versus* temperature. These results show that the variation of the on-resistance increases and the saturation current decreases with temperature. Note that the experimental results demonstrate the same behaviour as simulation one and hence agree with the proposed model.

## 5. Results and Discussion

**Figure 10.**
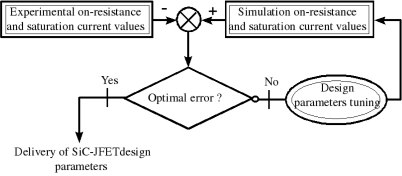
Flow char algorithm of SiC-JFETs design parameters identification.

In order to validate the proposed JFET sensor model, the following methodology is used. Firstly, experimental results are compared to the proposed model for both on-resistance and saturation current. Secondly, errors between the model and the experimental results are computed. Based on these errors, discrepancies between them are depicted in order to finally judge on the correction of the model parameters. Figure 10 summarize the previously presented steps respectively.

**Figure 11.**
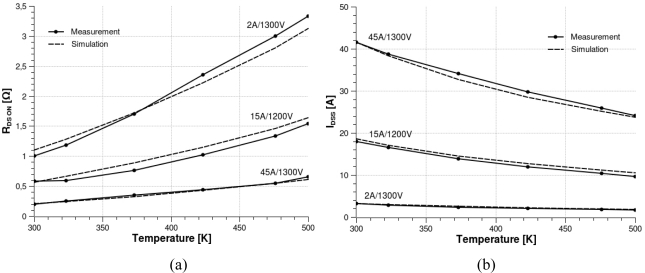
Comparison between experimental data and simulation results at various temperatures of three SiC-JFETs samples. (a) On-resistance and (b) saturation current.

Figures 11 (a and b) present a comparative study between simulation results and experimental data which are obtained for optimal design parameters depicted in table 1. Table 1 presents the design parameters for three SiC-JFETs. These parameters are obtained when the error between simulation and experimentation are optimal (flow char in Figure 10).

This flow char consists of two steps:

- Step#1: identifies (*N_DL_*, *N_DV_*, *N_sub_*, *W_drift_*, *h*, *L*, *Z*, *a* and *b*) parameters at ambient temperature according to on resistance (Equations 1–5) Then, the identified parameters are validated at different temperatures (Figure 11a).
- Step#2: comes after the step one, in order to validate the overall parameters at both ambient and different temperatures.

Important to note, that the estimated errors between the confidentional manufactured parameters and the obtained ones, are in good agreements. This paper presents, however, a simple approach to easy identification these parameters by only optimization error between experimental and simulation for on-resistance and saturation current at different temperatures level. This methodology may be applied for commercial or non commercial devices.

Table 1 presents the design and geometrical parameters identified for three SiC-JFETs samples. Optimal design parameters contribute to achieve simulation result precision as it is essential for sensor modelling and fabrication process. The validity of the model parameters given in Table 1 is obtained by a comparative study between simulation results and experimental data for many devices.

However, simulation errors cannot be avoided. They are calculated according to the on-resistance and the saturation current at different temperatures. An error less than 10% is found for the 2 A/1,300 V JFETs device sample, and less than 17% for the 15 A/1,200 V sample. These errors may be due to the non uniform doping concentration at the lateral and vertical channels. However, fabrication process is now mature enough and the error is less than 7% for the new 45 A/1,300 V sample.

In addition to its precision, the suggested model has got a good convergence. Furthermore, the simulation speed is significantly ameliorated: the model simulates in just 0.7 s on Pentium(R) Dual CPU (1.6 GHz) PC.

The ease of use is one more observed advantage. It consists in both the facility of implementation (VHDL-AMS language) and the simplicity in extracting model parameters (Figure 11). In addition, these parameters are technological and geometrical. Up to now literature has never identified the technological parameters of a SiC JFET. The design parameters are confidential and undocumented.

Moreover, the proposed algorithm, integrating the developed model, allows a better detection and adjustment of the SiC-JFET technological parameters (Figure 11). In fact, in the case of poor design and geometrical parameters, the model replies routinely by detecting and tuning it. Non-physical results and numerical problems are, therefore, methodically avoided.

## 6. Conclusionss

In this paper we have demonstrated that the two-channel SiC-JFETs have the on-resistance and the saturation current directly proportional to the absolute temperature. Therefore, we can use the SiC-JFET as a temperature sensor for hard environments on account of its robustness as well as semiconductor adequacy. A physically-based model is also proposed. The novel feature of this model is that it takes into account the design structure as well as technological parameters. The model validation is performed by comparing experimental results with simulation. This model is run for a large range temperature, and the simulation investigations show a good agreement with respect to the experimental ones. A systematic process enabling to evaluate JFET design parameters is also presented. These parameters are validated on several JFETs devices. Design parameters contribute to achieve simulation result accuracy as they are crucial for sensor modelling and fabrication process. Future works concern the electro-thermal validation of the JFET model and its application to recent 50 A/1700 V JFETs devices at 600 K.

## Figures and Tables

**Table 1.** Optimal design parameter set for three SiC JFETs samples.

	**2 A** **1300 V**	**15 A** **1200 V**	**45 A** **1300 V**
*a* [μm]	0.21	0.46	0.5
*b* [μm]	0.57	0.76	0.59
*L* [μm]	4.51	5.7	5.45
*h* [μm]	1.81	1.05	1.82
*N_DL_* [cm^−3^]	3 × 10^16^	5 × 10^16^	7 × 10^16^
*W_drift_* [μm]	8.12	7.5	7.43
*N_DV_* [cm^−3^]	1.2 × 10^16^	1.3 × 10^16^	1.34 × 10^16^
*L_drift_* [μm]	15	19	15
*Z* [cm]	4	3.8	10
